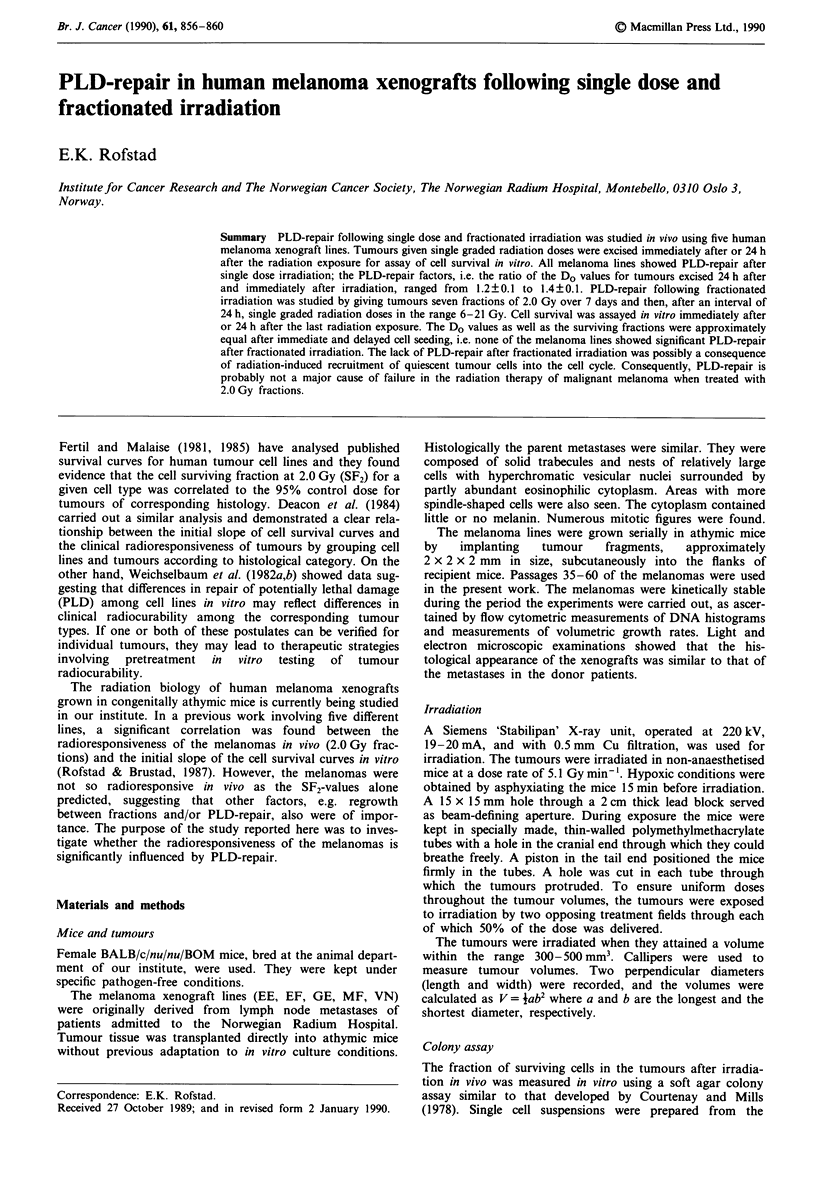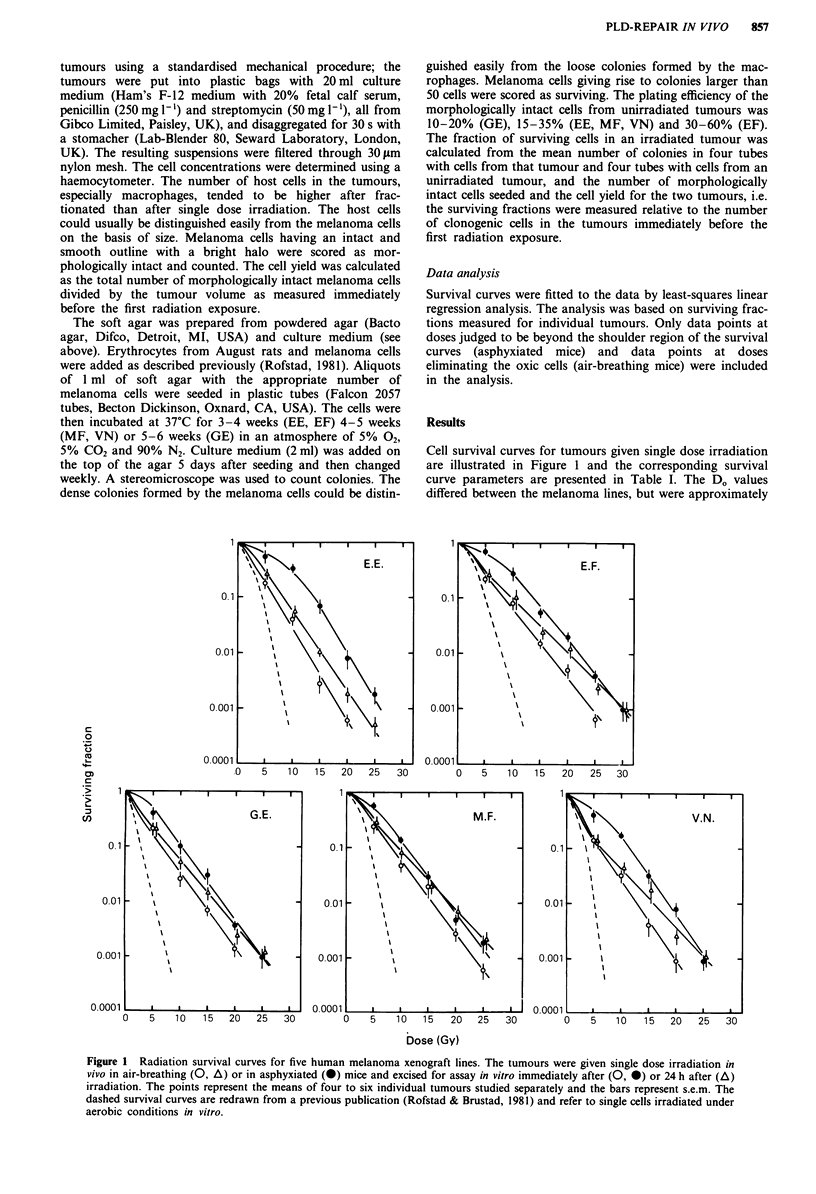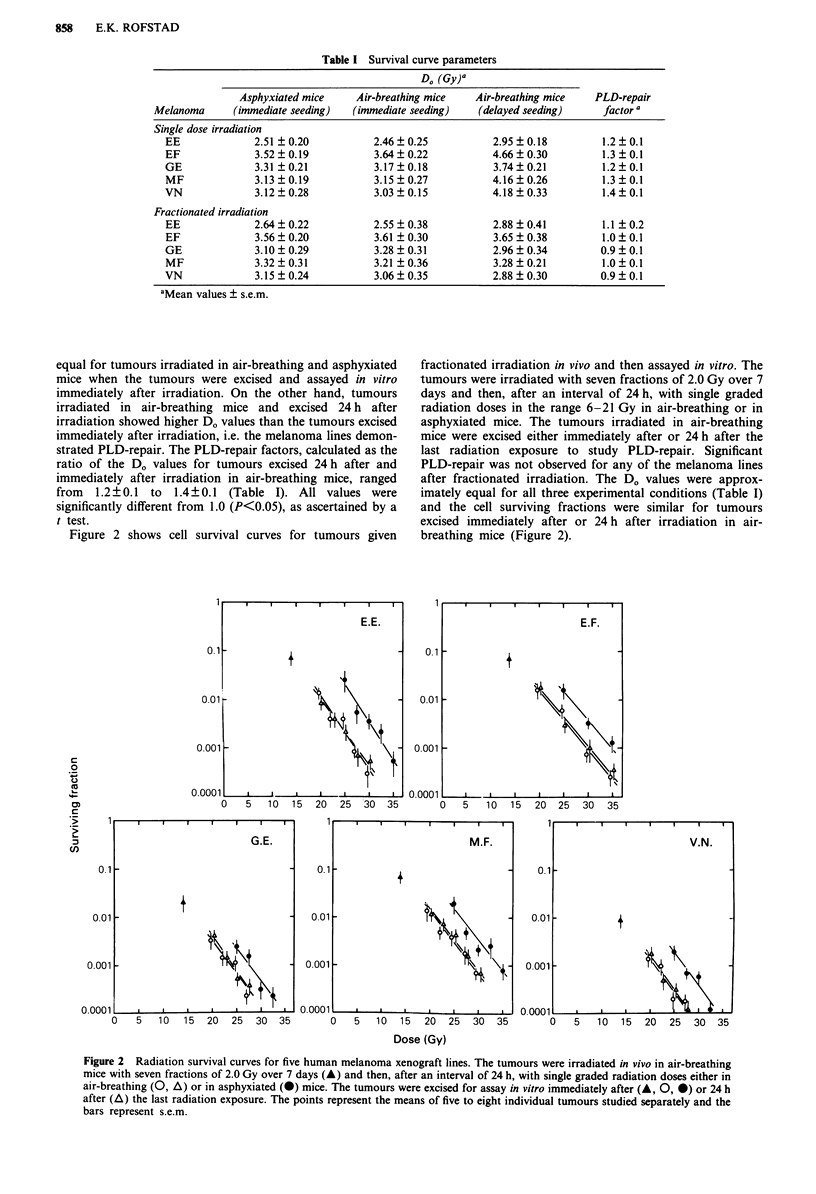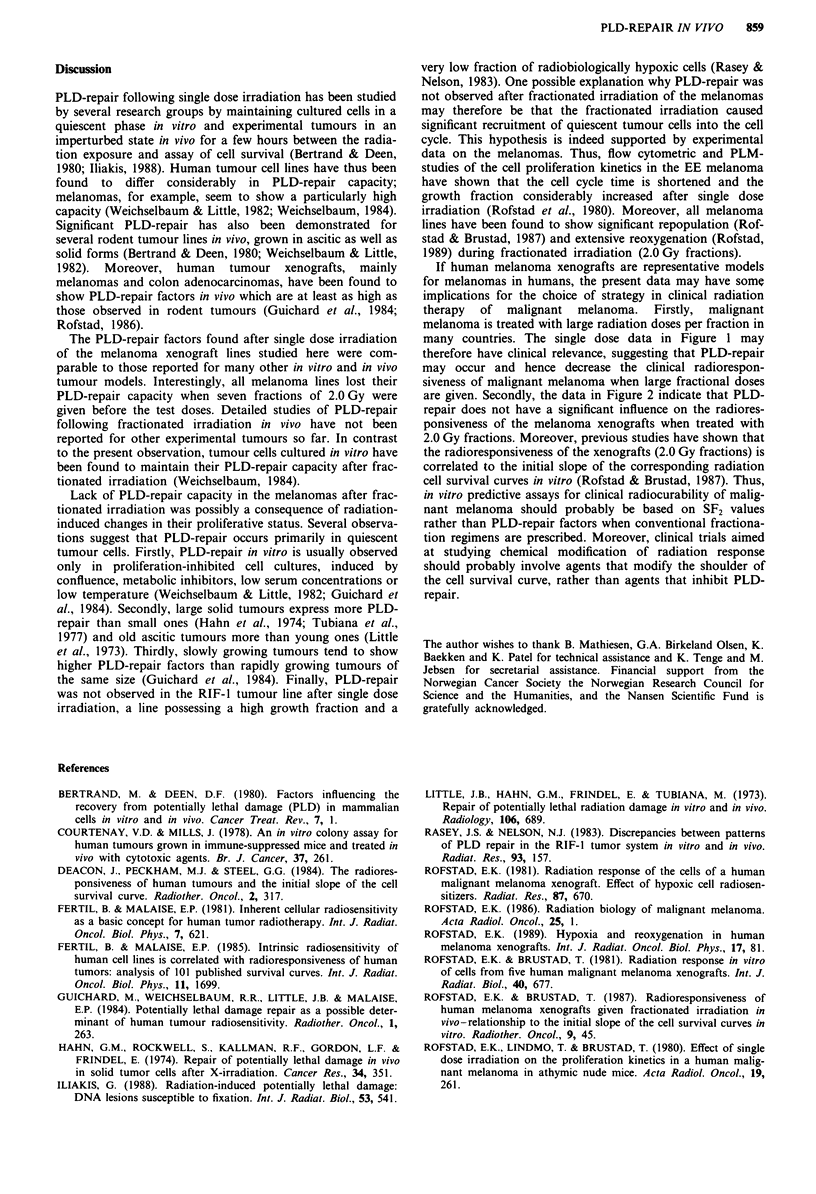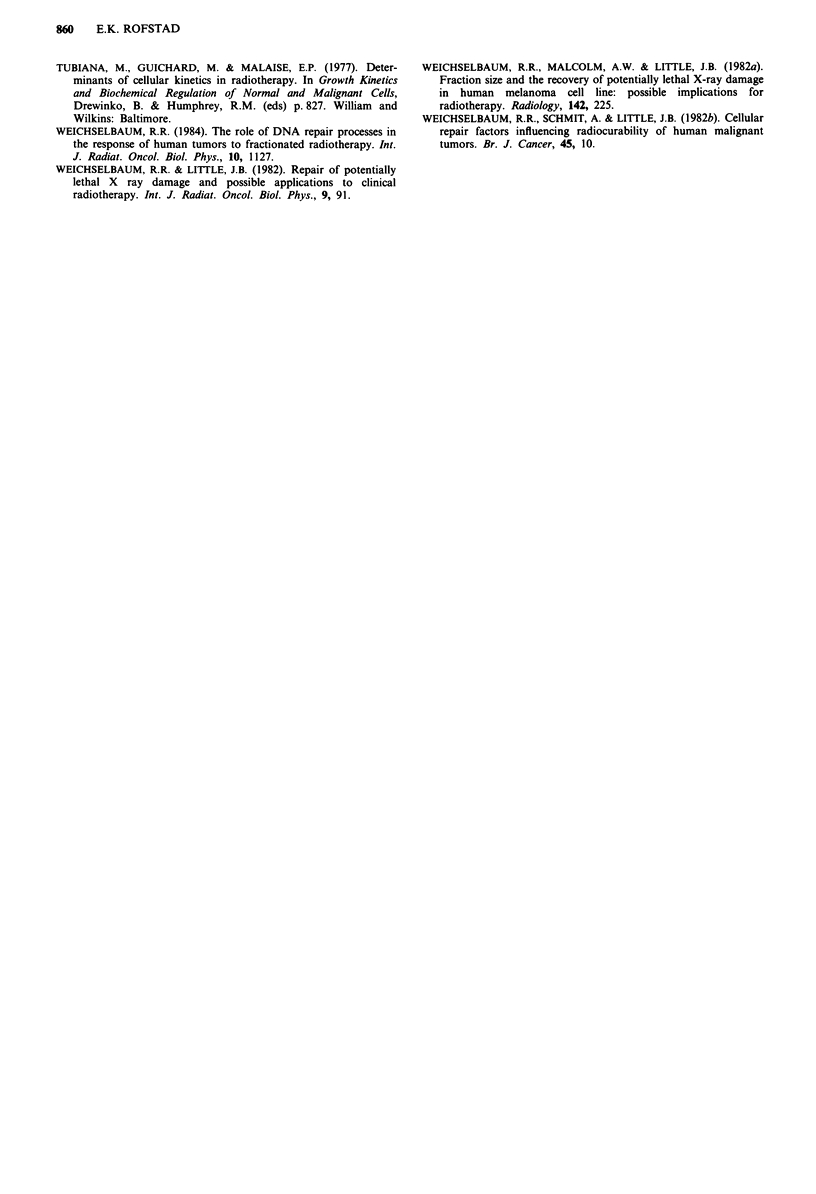# PLD-repair in human melanoma xenografts following single dose and fractionated irradiation.

**DOI:** 10.1038/bjc.1990.191

**Published:** 1990-06

**Authors:** E. K. Rofstad

**Affiliations:** Institute for Cancer Research, Norwegian Radium Hospital, Oslo.

## Abstract

PLD-repair following single dose and fractionated irradiation was studied in vivo using five human melanoma xenograft lines. Tumours given single graded radiation doses were excised immediately after or 24 h after the radiation exposure for assay of cell survival in vitro. All melanoma lines showed PLD-repair after single dose irradiation: the PLD-repair factors, i.e. the ratio of the Do values for tumours excised 24 h after and immediately after irradiation, ranged from 1.2 +/- 0.1 to 1.4 +/- 0.1. PLD-repair following fractionated irradiation was studied by giving tumours seven fractions of 2.0 Gy over 7 days and then, after an interval of 24 h, single graded radiation doses in the range 6-21 Gy. Cell survival was assayed in vitro immediately after or 24 h after the last radiation exposure. The Do values as well as the surviving fractions were approximately equal after immediate and delayed cell seeding, i.e. none of the melanoma lines showed significant PLD-repair after fractionated irradiation. The lack of PLD-repair after fractionated irradiation was possibly a consequence of radiation-induced recruitment of quiescent tumour cells into the cell cycle. Consequently, PLD-repair is probably not a major cause of failure in the radiation therapy of malignant melanoma when treated with 2.0 Gy fractions.


*Br. J. Cancer* (1990), **61**, 856–860 © Macmillan Press Ltd., 1990

### PLD-repair in human melanoma xenografts following single dose and fractionated irradiation

E.K. Rofstad

*Institute for Cancer Research and The Norwegian Cancer Society, The Norwegian Radium Hospital, Montebello, 0310 Oslo 3, Norway.*

**Summary** PLD-repair following single dose and fractionated irradiation was studied *in vivo* using five human melanoma xenograft lines. Tumours given single graded radiation doses were excised immediately after or 24 h after the radiation exposure for assay of cell survival *in vitro*. All melanoma lines showed PLD-repair after single dose irradiation; the PLD-repair factors, i.e. the ratio of the $D_0$ values for tumours excised 24 h after and immediately after irradiation, ranged from 1.2±0.1 to 1.4±0.1. PLD-repair following fractionated irradiation was studied by giving tumours seven fractions of 2.0 Gy over 7 days and then, after an interval of 24 h, single graded radiation doses in the range 6–21 Gy. Cell survival was assayed *in vitro* immediately after or 24 h after the last radiation exposure. The $D_0$ values as well as the surviving fractions were approximately equal after immediate and delayed cell seeding, i.e. none of the melanoma lines showed significant PLD-repair after fractionated irradiation. The lack of PLD-repair after fractionated irradiation was possibly a consequence of radiation-induced recruitment of quiescent tumour cells into the cell cycle. Consequently, PLD-repair is probably not a major cause of failure in the radiation therapy of malignant melanoma when treated with 2.0 Gy fractions.

Fertil and Malaise (1981, 1985) have analysed published survival curves for human tumour cell lines and they found evidence that the cell surviving fraction at 2.0 Gy ($SF_2$) for a given cell type was correlated to the 95% control dose for tumours of corresponding histology. Deacon *et al.* (1984) carried out a similar analysis and demonstrated a clear relationship between the initial slope of cell survival curves and the clinical radioresponsiveness of tumours by grouping cell lines and tumours according to histological category. On the other hand, Weichselbaum *et al.* (1982*a*,*b*) showed data suggesting that differences in repair of potentially lethal damage (PLD) among cell lines *in vitro* may reflect differences in clinical radiocurability among the corresponding tumour types. If one or both of these postulates can be verified for individual tumours, they may lead to therapeutic strategies involving pretreatment *in vitro* testing of tumour radiocurability.

The radiation biology of human melanoma xenografts grown in congenitally athymic mice is currently being studied in our institute. In a previous work involving five different lines, a significant correlation was found between the radioresponsiveness of the melanomas *in vivo* (2.0 Gy fractions) and the initial slope of the cell survival curves *in vitro* (Rofstad & Brustad, 1987). However, the melanomas were not so radioresponsive *in vivo* as the $SF_2$-values alone predicted, suggesting that other factors, e.g. regrowth between fractions and/or PLD-repair, also were of importance. The purpose of the study reported here was to investigate whether the radioresponsiveness of the melanomas is significantly influenced by PLD-repair.

### Materials and methods

### *Mice and tumours*

Female BALB/c/*nu*/*nu*/BOM mice, bred at the animal department of our institute, were used. They were kept under specific pathogen-free conditions.

The melanoma xenograft lines (EE, EF, GE, MF, VN) were originally derived from lymph node metastases of patients admitted to the Norwegian Radium Hospital. Tumour tissue was transplanted directly into athymic mice without previous adaptation to *in vitro* culture conditions. Histologically the parent metastases were similar. They were composed of solid trabecules and nests of relatively large cells with hyperchromatic vesicular nuclei surrounded by partly abundant eosinophilic cytoplasm. Areas with more spindle-shaped cells were also seen. The cytoplasm contained little or no melanin. Numerous mitotic figures were found.

The melanoma lines were grown serially in athymic mice by implanting tumour fragments, approximately 2 × 2 × 2 mm in size, subcutaneously into the flanks of recipient mice. Passages 35–60 of the melanomas were used in the present work. The melanomas were kinetically stable during the period the experiments were carried out, as ascertained by flow cytometric measurements of DNA histograms and measurements of volumetric growth rates. Light and electron microscopic examinations showed that the histological appearance of the xenografts was similar to that of the metastases in the donor patients.

### *Irradiation*

A Siemens 'Stabilipan' X-ray unit, operated at 220 kV, 19–20 mA, and with 0.5 mm Cu filtration, was used for irradiation. The tumours were irradiated in non-anaesthetised mice at a dose rate of 5.1 Gy $min^{-1}$. Hypoxic conditions were obtained by asphyxiating the mice 15 min before irradiation. A 15 × 15 mm hole through a 2 cm thick lead block served as beam-defining aperture. During exposure the mice were kept in specially made, thin-walled polymethylmethacrylate tubes with a hole in the cranial end through which they could breathe freely. A piston in the tail end positioned the mice firmly in the tubes. A hole was cut in each tube through which the tumours protruded. To ensure uniform doses throughout the tumour volumes, the tumours were exposed to irradiation by two opposing treatment fields through each of which 50% of the dose was delivered.

The tumours were irradiated when they attained a volume within the range 300–500 $mm^3$. Callipers were used to measure tumour volumes. Two perpendicular diameters (length and width) were recorded, and the volumes were calculated as $V = \frac{1}{2}ab^2$ where $a$ and $b$ are the longest and the shortest diameter, respectively.

### *Colony assay*

The fraction of surviving cells in the tumours after irradiation *in vivo* was measured *in vitro* using a soft agar colony assay similar to that developed by Courtenay and Mills (1978). Single cell suspensions were prepared from the

Correspondence: E.K. Rofstad.
Received 27 October 1989; and in revised form 2 January 1990.

tumours using a standardised mechanical procedure; the tumours were put into plastic bags with 20 ml culture medium (Ham's F-12 medium with 20% fetal calf serum, penicillin (250 mg $l^{-1}$) and streptomycin (50 mg $l^{-1}$), all from Gibco Limited, Paisley, UK), and disaggregated for 30 s with a stomacher (Lab-Blender 80, Seward Laboratory, London, UK). The resulting suspensions were filtered through 30 μm nylon mesh. The cell concentrations were determined using a haemocytometer. The number of host cells in the tumours, especially macrophages, tended to be higher after fractionated than after single dose irradiation. The host cells could usually be distinguished easily from the melanoma cells on the basis of size. Melanoma cells having an intact and smooth outline with a bright halo were scored as morphologically intact and counted. The cell yield was calculated as the total number of morphologically intact melanoma cells divided by the tumour volume as measured immediately before the first radiation exposure.

The soft agar was prepared from powdered agar (Bacto agar, Difco, Detroit, MI, USA) and culture medium (see above). Erythrocytes from August rats and melanoma cells were added as described previously (Rofstad, 1981). Aliquots of 1 ml of soft agar with the appropriate number of melanoma cells were seeded in plastic tubes (Falcon 2057 tubes, Becton Dickinson, Oxnard, CA, USA). The cells were then incubated at 37°C for 3–4 weeks (EE, EF) 4–5 weeks (MF, VN) or 5–6 weeks (GE) in an atmosphere of 5% $O_2$, 5% $CO_2$ and 90% $N_2$. Culture medium (2 ml) was added on the top of the agar 5 days after seeding and then changed weekly. A stereomicroscope was used to count colonies. The dense colonies formed by the melanoma cells could be distinguished easily from the loose colonies formed by the macrophages. Melanoma cells giving rise to colonies larger than 50 cells were scored as surviving. The plating efficiency of the morphologically intact cells from unirradiated tumours was 10–20% (GE), 15–35% (EE, MF, VN) and 30–60% (EF). The fraction of surviving cells in an irradiated tumour was calculated from the mean number of colonies in four tubes with cells from that tumour and four tubes with cells from an unirradiated tumour, and the number of morphologically intact cells seeded and the cell yield for the two tumours, i.e. the surviving fractions were measured relative to the number of clonogenic cells in the tumours immediately before the first radiation exposure.

### *Data analysis*

Survival curves were fitted to the data by least-squares linear regression analysis. The analysis was based on surviving fractions measured for individual tumours. Only data points at doses judged to be beyond the shoulder region of the survival curves (asphyxiated mice) and data points at doses eliminating the oxic cells (air-breathing mice) were included in the analysis.

### Results

Cell survival curves for tumours given single dose irradiation are illustrated in Figure 1 and the corresponding survival curve parameters are presented in Table I. The $D_0$ values differed between the melanoma lines, but were approximately

**Figure 1** Radiation survival curves for five human melanoma xenograft lines. The tumours were given single dose irradiation *in vivo* in air-breathing (O, Δ) or in asphyxiated (●) mice and excised for assay *in vitro* immediately after (O, ●) or 24 h after (Δ) irradiation. The points represent the means of four to six individual tumours studied separately and the bars represent s.e.m. The dashed survival curves are redrawn from a previous publication (Rofstad & Brustad, 1981) and refer to single cells irradiated under aerobic conditions *in vitro*.

**Table I** Survival curve parameters

| Melanoma | $D_0$ (Gy)[a] Asphyxiated mice (immediate seeding) | Air-breathing mice (immediate seeding) | Air-breathing mice (delayed seeding) | PLD-repair factor[a] |
|---|---|---|---|---|
| *Single dose irradiation* | | | | |
| EE | 2.51 ± 0.20 | 2.46 ± 0.25 | 2.95 ± 0.18 | 1.2 ± 0.1 |
| EF | 3.52 ± 0.19 | 3.64 ± 0.22 | 4.66 ± 0.30 | 1.3 ± 0.1 |
| GE | 3.31 ± 0.21 | 3.17 ± 0.18 | 3.74 ± 0.21 | 1.2 ± 0.1 |
| MF | 3.13 ± 0.19 | 3.15 ± 0.27 | 4.16 ± 0.26 | 1.3 ± 0.1 |
| VN | 3.12 ± 0.28 | 3.03 ± 0.15 | 4.18 ± 0.33 | 1.4 ± 0.1 |
| *Fractionated irradiation* | | | | |
| EE | 2.64 ± 0.22 | 2.55 ± 0.38 | 2.88 ± 0.41 | 1.1 ± 0.2 |
| EF | 3.56 ± 0.20 | 3.61 ± 0.30 | 3.65 ± 0.38 | 1.0 ± 0.1 |
| GE | 3.10 ± 0.29 | 3.28 ± 0.31 | 2.96 ± 0.34 | 0.9 ± 0.1 |
| MF | 3.32 ± 0.31 | 3.21 ± 0.36 | 3.28 ± 0.21 | 1.0 ± 0.1 |
| VN | 3.15 ± 0.24 | 3.06 ± 0.35 | 2.88 ± 0.30 | 0.9 ± 0.1 |

[a]Mean values ± s.e.m.

equal for tumours irradiated in air-breathing and asphyxiated mice when the tumours were excised and assayed *in vitro* immediately after irradiation. On the other hand, tumours irradiated in air-breathing mice and excised 24 h after irradiation showed higher $D_0$ values than the tumours excised immediately after irradiation, i.e. the melanoma lines demonstrated PLD-repair. The PLD-repair factors, calculated as the ratio of the $D_0$ values for tumours excised 24 h after and immediately after irradiation in air-breathing mice, ranged from 1.2±0.1 to 1.4±0.1 (Table I). All values were significantly different from 1.0 ($P<0.05$), as ascertained by a *t* test.

Figure 2 shows cell survival curves for tumours given fractionated irradiation *in vivo* and then assayed *in vitro*. The tumours were irradiated with seven fractions of 2.0 Gy over 7 days and then, after an interval of 24 h, with single graded radiation doses in the range 6–21 Gy in air-breathing or in asphyxiated mice. The tumours irradiated in air-breathing mice were excised either immediately after or 24 h after the last radiation exposure to study PLD-repair. Significant PLD-repair was not observed for any of the melanoma lines after fractionated irradiation. The $D_0$ values were approximately equal for all three experimental conditions (Table I) and the cell surviving fractions were similar for tumours excised immediately after or 24 h after irradiation in air-breathing mice (Figure 2).

**Figure 2** Radiation survival curves for five human melanoma xenograft lines. The tumours were irradiated *in vivo* in air-breathing mice with seven fractions of 2.0 Gy over 7 days (▲) and then, after an interval of 24 h, with single graded radiation doses either in air-breathing (○, △) or in asphyxiated (●) mice. The tumours were excised for assay *in vitro* immediately after (▲, ○, ●) or 24 h after (△) the last radiation exposure. The points represent the means of five to eight individual tumours studied separately and the bars represent s.e.m.

### Discussion

PLD-repair following single dose irradiation has been studied by several research groups by maintaining cultured cells in a quiescent phase *in vitro* and experimental tumours in an imperturbed state *in vivo* for a few hours between the radiation exposure and assay of cell survival (Bertrand & Deen, 1980; Iliakis, 1988). Human tumour cell lines have thus been found to differ considerably in PLD-repair capacity; melanomas, for example, seem to show a particularly high capacity (Weichselbaum & Little, 1982; Weichselbaum, 1984). Significant PLD-repair has also been demonstrated for several rodent tumour lines *in vivo*, grown in ascitic as well as solid forms (Bertrand & Deen, 1980; Weichselbaum & Little, 1982). Moreover, human tumour xenografts, mainly melanomas and colon adenocarcinomas, have been found to show PLD-repair factors *in vivo* which are at least as high as those observed in rodent tumours (Guichard *et al.*, 1984; Rofstad, 1986).

The PLD-repair factors found after single dose irradiation of the melanoma xenograft lines studied here were comparable to those reported for many other *in vitro* and *in vivo* tumour models. Interestingly, all melanoma lines lost their PLD-repair capacity when seven fractions of 2.0 Gy were given before the test doses. Detailed studies of PLD-repair following fractionated irradiation *in vivo* have not been reported for other experimental tumours so far. In contrast to the present observation, tumour cells cultured *in vitro* have been found to maintain their PLD-repair capacity after fractionated irradiation (Weichselbaum, 1984).

Lack of PLD-repair capacity in the melanomas after fractionated irradiation was possibly a consequence of radiation-induced changes in their proliferative status. Several observations suggest that PLD-repair occurs primarily in quiescent tumour cells. Firstly, PLD-repair *in vitro* is usually observed only in proliferation-inhibited cell cultures, induced by confluence, metabolic inhibitors, low serum concentrations or low temperature (Weichselbaum & Little, 1982; Guichard *et al.*, 1984). Secondly, large solid tumours express more PLD-repair than small ones (Hahn *et al.*, 1974; Tubiana *et al.*, 1977) and old ascitic tumours more than young ones (Little *et al.*, 1973). Thirdly, slowly growing tumours tend to show higher PLD-repair factors than rapidly growing tumours of the same size (Guichard *et al.*, 1984). Finally, PLD-repair was not observed in the RIF-1 tumour line after single dose irradiation, a line possessing a high growth fraction and a very low fraction of radiobiologically hypoxic cells (Rasey & Nelson, 1983). One possible explanation why PLD-repair was not observed after fractionated irradiation of the melanomas may therefore be that the fractionated irradiation caused significant recruitment of quiescent tumour cells into the cell cycle. This hypothesis is indeed supported by experimental data on the melanomas. Thus, flow cytometric and PLM-studies of the cell proliferation kinetics in the EE melanoma have shown that the cell cycle time is shortened and the growth fraction considerably increased after single dose irradiation (Rofstad *et al.*, 1980). Moreover, all melanoma lines have been found to show significant repopulation (Rofstad & Brustad, 1987) and extensive reoxygenation (Rofstad, 1989) during fractionated irradiation (2.0 Gy fractions).

If human melanoma xenografts are representative models for melanomas in humans, the present data may have some implications for the choice of strategy in clinical radiation therapy of malignant melanoma. Firstly, malignant melanoma is treated with large radiation doses per fraction in many countries. The single dose data in Figure 1 may therefore have clinical relevance, suggesting that PLD-repair may occur and hence decrease the clinical radioresponsiveness of malignant melanoma when large fractional doses are given. Secondly, the data in Figure 2 indicate that PLD-repair does not have a significant influence on the radioresponsiveness of the melanoma xenografts when treated with 2.0 Gy fractions. Moreover, previous studies have shown that the radioresponsiveness of the xenografts (2.0 Gy fractions) is correlated to the initial slope of the corresponding radiation cell survival curves *in vitro* (Rofstad & Brustad, 1987). Thus, *in vitro* predictive assays for clinical radiocurability of malignant melanoma should probably be based on $SF_2$ values rather than PLD-repair factors when conventional fractionation regimens are prescribed. Moreover, clinical trials aimed at studying chemical modification of radiation response should probably involve agents that modify the shoulder of the cell survival curve, rather than agents that inhibit PLD-repair.

The author wishes to thank B. Mathiesen, G.A. Birkeland Olsen, K. Baekken and K. Patel for technical assistance and K. Tenge and M. Jebsen for secretarial assistance. Financial support from the Norwegian Cancer Society the Norwegian Research Council for Science and the Humanities, and the Nansen Scientific Fund is gratefully acknowledged.

### References

BERTRAND, M. & DEEN, D.F. (1980). Factors influencing the recovery from potentially lethal damage (PLD) in mammalian cells *in vitro* and *in vivo*. *Cancer Treat. Rev.*, **7**, 1.

COURTENAY, V.D. & MILLS, J. (1978). An *in vitro* colony assay for human tumours grown in immune-suppressed mice and treated *in vivo* with cytotoxic agents. *Br. J. Cancer*, **37**, 261.

DEACON, J., PECKHAM, M.J. & STEEL, G.G. (1984). The radioresponsiveness of human tumours and the initial slope of the cell survival curve. *Radiother. Oncol.*, **2**, 317.

FERTIL, B. & MALAISE, E.P. (1981). Inherent cellular radiosensitivity as a basic concept for human tumor radiotherapy. *Int. J. Radiat. Oncol. Biol. Phys.*, **7**, 621.

FERTIL, B. & MALAISE, E.P. (1985). Intrinsic radiosensitivity of human cell lines is correlated with radioresponsiveness of human tumors: analysis of 101 published survival curves. *Int. J. Radiat. Oncol. Biol. Phys.*, **11**, 1699.

GUICHARD, M., WEICHSELBAUM, R.R., LITTLE, J.B. & MALAISE, E.P. (1984). Potentially lethal damage repair as a possible determinant of human tumour radiosensitivity. *Radiother. Oncol.*, **1**, 263.

HAHN, G.M., ROCKWELL, S., KALLMAN, R.F., GORDON, L.F. & FRINDEL, E. (1974). Repair of potentially lethal damage *in vivo* in solid tumor cells after X-irradiation. *Cancer Res.*, **34**, 351.

ILIAKIS, G. (1988). Radiation-induced potentially lethal damage: DNA lesions susceptible to fixation. *Int. J. Radiat. Biol.*, **53**, 541.

LITTLE, J.B., HAHN, G.M., FRINDEL, E. & TUBIANA, M. (1973). Repair of potentially lethal radiation damage *in vitro* and *in vivo*. *Radiology*, **106**, 689.

RASEY, J.S. & NELSON, N.J. (1983). Discrepancies between patterns of PLD repair in the RIF-1 tumor system *in vitro* and *in vivo*. *Radiat. Res.*, **93**, 157.

ROFSTAD, E.K. (1981). Radiation response of the cells of a human malignant melanoma xenograft. Effect of hypoxic cell radiosensitizers. *Radiat. Res.*, **87**, 670.

ROFSTAD, E.K. (1986). Radiation biology of malignant melanoma. *Acta Radiol. Oncol.*, **25**, 1.

ROFSTAD, E.K. (1989). Hypoxia and reoxygenation in human melanoma xenografts. *Int. J. Radiat. Oncol. Biol. Phys.*, **17**, 81.

ROFSTAD, E.K. & BRUSTAD, T. (1981). Radiation response *in vitro* of cells from five human malignant melanoma xenografts. *Int. J. Radiat. Biol.*, **40**, 677.

ROFSTAD, E.K. & BRUSTAD, T. (1987). Radioresponsiveness of human melanoma xenografts given fractionated irradiation *in vivo*–relationship to the initial slope of the cell survival curves *in vitro*. *Radiother. Oncol.*, **9**, 45.

ROFSTAD, E.K., LINDMO, T. & BRUSTAD, T. (1980). Effect of single dose irradiation on the proliferation kinetics in a human malignant melanoma in athymic nude mice. *Acta Radiol. Oncol.*, **19**, 261.

TUBIANA, M., GUICHARD, M. & MALAISE, E.P. (1977). Determinants of cellular kinetics in radiotherapy. In *Growth Kinetics and Biochemical Regulation of Normal and Malignant Cells*, Drewinko, B. & Humphrey, R.M. (eds) p. 827. William and Wilkins: Baltimore.

WEICHSELBAUM, R.R. (1984). The role of DNA repair processes in the response of human tumors to fractionated radiotherapy. *Int. J. Radiat. Oncol. Biol. Phys.*, **10**, 1127.

WEICHSELBAUM, R.R. & LITTLE, J.B. (1982). Repair of potentially lethal X ray damage and possible applications to clinical radiotherapy. *Int. J. Radiat. Oncol. Biol. Phys.*, **9**, 91.

WEICHSELBAUM, R.R., MALCOLM, A.W. & LITTLE, J.B. (1982*a*). Fraction size and the recovery of potentially lethal X-ray damage in human melanoma cell line: possible implications for radiotherapy. *Radiology*, **142**, 225.

WEICHSELBAUM, R.R., SCHMIT, A. & LITTLE, J.B. (1982*b*). Cellular repair factors influencing radiocurability of human malignant tumors. *Br. J. Cancer*, **45**, 10.